# Analgesic strategies for ischaemic pain in chronic limb–threatening ischaemia: a systematic review and meta-analysis

**DOI:** 10.1097/PR9.0000000000001426

**Published:** 2026-03-10

**Authors:** Henry Davies, Ahmed Boalot, Annabel Howitt, Marie-José Vleugels, Sharon Ka Po Tam, Jing Yi Kwan, Sheila Black, Ingrid Francis, Christopher Bull, Barend M.E. Mees, Sarah Mitchell, David Russell

**Affiliations:** a University of Leeds, Leeds, United Kingdom; b Calderdale and Huddersfield NHS Foundation Trust, Huddersfield, United Kingdom; c Leeds Teaching Hospitals NHS Trust, Leeds, United Kingdom; d Department of Vascular Surgery, Maastricht UMC+, Maastricht, Netherlands; e Bradford Teaching Hospitals NHS Trust, Bradford, United Kingdom

**Keywords:** Chronic limb–threatening ischaemia, Ischaemic pain, Analgesia, Pain management, Peripheral artery disease, Systematic review, Meta-analysis

## Abstract

To evaluate the effectiveness of pharmacological (eg, ketamine, lidocaine) and nonpharmacological (eg, spinal cord stimulation, mononuclear cell therapy) analgesic strategies—outside the WHO pain ladder—for patients with chronic limb–threatening ischaemia (CLTI). A systematic review and meta-analysis was conducted in accordance with PRISMA guidelines, searching 5 electronic databases for studies evaluating analgesia in CLTI. Both randomised and nonrandomised studies were included. Standardised mean differences (SMDs) in post-treatment pain scores were extracted or calculated, and random-effects meta-analyses performed for comparable studies. Twenty-one studies met inclusion criteria. Most investigated mononuclear cell therapy, hepatocyte growth factor, or prostaglandin infusions. Meta-analyses of mononuclear cell therapy vs standard treatment at 3 months showed significant benefit (3 studies; SMD = −2.12, 95% confidence interval [CI] −3.19 to −1.05, *P* = 0.001, I^2^ = 86.95%). At 6 months, benefit persisted (3 studies; SMD = −2.54, 95% CI −3.95 to −1.14, *P* = 0.0004, I^2^ = 92.0%). Across studies, mononuclear cell therapy showed the largest effect sizes but was associated with high heterogeneity and, due to restrictive exclusion criteria, had limited generalisability and poor practicality. Hepatocyte growth factor therapy showed a nonsignificant trend towards pain reduction, and prostaglandin infusion demonstrated no meaningful effect; both excluded large proportions of the CLTI cohort. Of the single-study interventions, subcutaneous lidocaine and ketamine provided short-term analgesic benefit with acceptable safety, whereas others did not produce statistically significant improvements. Pharmacological management of pain in CLTI remains challenging. No recommendations can be made for agents outside standard care. Several novel approaches, including ketamine and subcutaneous lidocaine, warrant further investigation. Further research into novel analgesics for CLTI is urgently needed.

## Abstract 2

Supplemental Digital Content is Available in the Text.

Evidence for treating ischaemic pain in chronic limb–threatening ischaemia is limited, and no recommendations can be made following this systematic review of the evidence.

## 1. Introduction

Chronic limb–threatening ischaemia (CLTI), the end stage of peripheral artery disease, is characterised by an insufficient blood supply leading to a debilitating rest pain (with or without tissue loss), which significantly impairs patient quality of life.^7,43^ Chronic limb–threatening ischaemia patients experience severe pain that is difficult to manage with standard analgesics. This is in part due to the complex underlying mechanisms inherent to ischaemic pain.^47^ In 1986, the World Health Organization (WHO) introduced the pain ladder, drawing primarily on evidence from cancer-related pain, and was intended for the management of this type of pain.^45^ Despite its original design being for the management of oncological pain, the ladder has since been widely adopted for the management of all pain, despite the fact that most patients do not have cancer. This extrapolation has occurred without robust evidence supporting its applicability to many noncancer pain syndromes. In particular, ischaemic pain in CLTI has distinct pathophysiological mechanisms, including neuropathic and inflammatory components, which may limit responsiveness to the stepwise escalation of opioids and adjuvants advocated by the WHO ladder. Its primary origin lies in tissue hypoxia and metabolic dysfunction, where insufficient arterial perfusion causes metabolic byproducts like lactate, adenosine, and potassium ions to build up, directly activating nociceptors in arterial walls and ischaemic tissues, producing a characteristic deep, aching pain exacerbated by dependency.^12,37^ Superimposed on this nociceptive component is a significant neuropathic component, resulting from chronic ischaemia–induced nerve damage. Prolonged hypoxia causes axonal degeneration in both somatic and autonomic nerve fibres, resulting in abnormal spontaneous activity that presents as burning sensations, allodynia and hyperalgesia.^2,5^ Furthermore, ischaemia compromises the blood–nerve barrier, allowing inflammatory mediators to infiltrate neural tissues and worsen nerve dysfunction.^40^ Inflammation further complicates pain by activating immune cells and releasing proinflammatory cytokines such as tumour necrosis factor-α, interleukin-1β, and interleukin-6.^46,49^ These cytokines directly sensitise nociceptors and promote the recruitment of additional inflammatory cells, thereby amplifying the inflammatory response via nuclear factor-kappa B pathway activation.^31^ Ischaemia–reperfusion injury can further exacerbate this inflammatory cascade through the sudden restoration of oxygen, which paradoxically increases tissue damage via reactive oxygen species generation.^14,23^ Although the degree of ischaemia correlates with pain severity, it does not fully predict it, highlighting the importance of central pain processing mechanisms and individual variations in pain sensitivity.^48^

Despite the mechanisms of the nociceptive and neuropathic pain pathways being relatively well understood, the interplay between them and other pathways involved in the manifestation of ischaemic pain in CLTI is considerably less well understood.^20^ This multifactorial nature of ischaemic pain poses significant therapeutic challenges for conventional analgesics. The pain experience is highly variable, influenced by factors such as tissue viability, anatomical location, and ischaemia severity, with individual patients exhibiting varying degrees of the nociceptive, neuropathic, and inflammatory components.^21,41^ This heterogeneity suggests that a single therapeutic approach is unlikely to provide adequate relief for these diverse, simultaneously operating, pain mechanisms.

Current pain management strategies for CLTI are limited by complex pathophysiology and multiple comorbidities, which limit available therapeutic options.^21^ The absence of standardised, evidence-based protocols for CLTI pain management contributes to significant practice variation and suboptimal outcomes.^13^

Evaluation of pharmacological and interventional analgesic strategies for CLTI pain is a critical research priority given the substantial burden of suffering and the limitations of current approaches.^4^ The unique pathophysiological characteristics of ischaemic pain necessitate targeted research that cannot be extrapolated from other chronic pain conditions.^38^ Effective pain control may facilitate wound healing, improve functional outcomes, and enhance quality of life while potentially reducing health care utilisation.^22^ This systematic review synthesises current evidence on analgesic strategies for ischaemic pain in CLTI, assessing its capacity to inform clinical decision making and highlight priorities for future research.

## 2. Methods

### 2.1. Protocol and registration

This review was conducted following the Preferred Reporting Items for Systematic Reviews and Meta-Analyses (PRISMA) guidelines.^33^ The protocol was prospectively registered with the International Prospective Register of Systematic Reviews (PROSPERO; CRD42025641638)^36^ on January 29, 2025.

### 2.2. Eligibility criteria

Studies between 2000 and 2025 were included if they met the following criteria: participants were adults aged 18 years or older with CLTI; interventions included any pharmacological or nonpharmacological treatment aimed at relieving ischaemic pain, including both combination therapies and monotherapies; comparators included placebo, standard analgesic regimens, or alternative pharmacological treatments; and outcomes included measures of pain relief or related clinical endpoints.

Studies were excluded if they involved experimental animal models or healthy volunteers with induced ischaemic pain. No restrictions were placed on study design, publication date, or language to ensure comprehensive inclusion of global evidence. The population of interest specifically comprised patients experiencing intractable pain from ongoing ischaemia despite conventional analgesia, representing those for whom standard pain management approaches had proven insufficient. Standard pain management for the purpose of this study was defined as analgesia included in the WHO pain ladder.

### 2.3. Information sources and search strategy

We searched MEDLINE, EMBASE, the Cochrane Library, Web of Science, and CINAHL from 2000 to 2025, without language restrictions. The strategy combined indexed terms and free-text keywords for “analgesia,” “ischaemic pain,” and “chronic limb threatening ischaemia,” adapted for each database. Additional sources included reference lists of eligible studies and relevant reviews, as well as direct contact with authors and experts in the field. The search strategy is shown in Supplementary Figure 1, http://links.lww.com/PR9/A392.

### 2.4. Study selection

**Figure 1.**
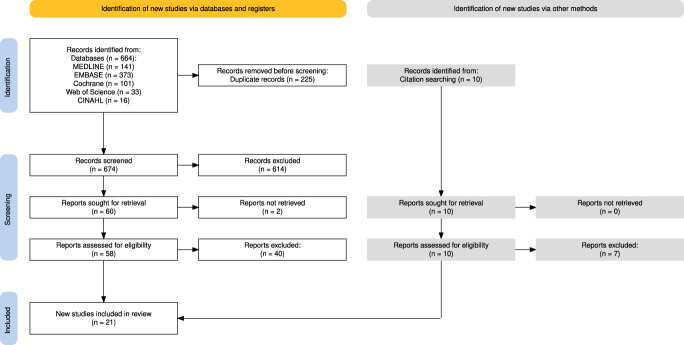
PRISMA flow diagram of study selection. Flow diagram illustrating the process of study identification, screening, eligibility assessment, and inclusion, in accordance with the PRISMA guidelines. Numbers of records identified through database searching and other sources, records screened, full-text articles assessed for eligibility, and studies included are shown. PRISMA, preferred reporting items for systematic reviews and meta-analyses.

Two reviewers (H.D. and A.H.) independently screened all records in Rayyan.^6^ Titles and abstracts were checked first, followed by full-text review of potentially eligible studies. Disagreements were resolved by discussion or a third reviewer (D.R.). The PRISMA flow diagram is shown in Figure 1.^35^ A total of 21 studies were identified.

### 2.5. Data extraction

Two reviewers (H.D. and A.B.) independently extracted data into a predesigned spreadsheet. Extracted items included study details (ID, authors, year, country, design, setting, sample size), participant characteristics (comorbidities, baseline pain scores), intervention and control details (treatment type, dosage, duration, analgesic used), outcomes (pre- and post-treatment pain scores, standard deviations, change scores, timepoints, pain scale type and range), and methodological information (risk of bias domains). Discrepancies were resolved by discussion or by a third reviewer (D.R.).

### 2.6. Risk of bias assessment

Risk of bias was assessed independently by 2 reviewers using the Cochrane Risk of Bias Tool (RoB 2) for randomised controlled trials (RCTs) and the Newcastle–Ottawa Scale (NOS) tool for nonrandomised studies. Both low- and high-risk studies were included, with sensitivity analyses planned to assess their influence on the findings if data sufficed.

### 2.7. Data synthesis

Where sufficient homogeneity was present, meta-analyses were conducted in R (R version 4.1.2; R Foundation for Statistical Computing, Vienna, Austria) using the meta and metafor packages. Analyses used a random-effects model, chosen a priori to account for expected clinical and methodological heterogeneity between studies in populations, interventions, outcome measures, and follow-up durations.

Given the substantial heterogeneity in how pain outcomes were reported across studies (eg, categorical improvements, change scores without standard deviations, or outcomes at unmatched timepoints), quantitative synthesis was restricted to trials reporting *continuous post-treatment pain scores with complete variance data* (mean ± SD) for both treatment and control groups at harmonised timepoints. This restriction ensured appropriate and consistent calculation of standardised mean differences. All other interventions were synthesised narratively in accordance with the prespecified protocol.

Continuous outcomes were summarised as standardised mean differences (SMDs) with 95% confidence intervals (CIs). Binary outcomes were summarised as pooled log risk ratios with 95% CIs. Heterogeneity was assessed using the I^2^ statistic.

In accordance with current guidance, assessments of publication bias did not include Egger regression test due to the small number of studies (<10) available for each meta-analysis. Funnel plots were inspected visually only.

## 3. Results

### 3.1. Study characteristics

Twenty-one studies^1,3,9–11,15,17,18,24,25,27–30,32,34,39,42,44^ met inclusion criteria (Table 1), comprising 19 randomised controlled trials^1,3,9–11,15,16,18,24,25,27–30,32,34,39,42,44^ and 2 nonrandomised cohort studies.^8,19^ Trials were conducted across 13 countries, with the most frequent settings being Europe (n = 13) and Asia (n = 6). Twelve studies were multicentre, and 9 were single-centre.

**Table 1:** Characteristics of included studies evaluating interventions for ischaemic pain in chronic limb–threatening ischaemia.

Study	Country	Groups	Intervention	Control	Design	Centre	Sample size (treatment)	Sample size (control)	Follow-up duration	Pain outcome
Anghel et al.^1^ (2011)	Romania	HGF vs standard treatment	Vascular endothelial and hepatocyte growth factor gene	Plasmids alone	RCT (double blind)	Multi	29	14	3 mo and 6 mo	Binary
Di et al.^10^ (2022)	China	HGF vs standard treatment	Recombinant human hepatocyte growth factor plasmid	Normal saline	RCT (double blind)	Multi	360	180	3 mo and 6 mo	NRS
Gu et al.^17^ (2021)	China	HGF vs standard treatment	Human hepatocyte growth factor plasmid	Normal saline	RCT (double blind)	Multi	25	29	3 mo and 6 mo	VAS
Powell et al.^34^ (2008)	USA	HGF vs standard treatment	Human hepatocyte growth factor plasmid	Normal saline	RCT (double blind)	Multi	27	26	3 mo and 6 mo	VAS
Mitchell and Fallon^29^ (2002)	United Kingdom	Ket vs standard treatment	Intravenous low-dose ketamine	Normal saline	RCT (double blind)	Multi	18	17	Less than 2 wk	Likert
De Angelis et al.^8^ (2015)	Italy	MNC vs standard treatment	Autologous peripheral blood mononuclear cells	Conventional therapies	Cohort Study	Single	43	43	3 mo and 6 mo	VAS
Gupta et al.^18^ (2013)	India	MNC vs standard treatment	Bone marrow–derived mesenchymal stem cells	PlasmaLyte A	RCT (double blind)	Multi	10	10	3 mo and 6 mo	VAS
Gupta et al.^19^ (2017)	India	MNC vs standard treatment	Bone marrow–derived mesenchymal stem cells	Standard of care	Cohort Study	Multi	36	18	3 mo and 6 mo	VAS
Lu et al.^28^ (2011)	China	MNC vs standard treatment	Bone marrow mesenchymal stem cells	Normal saline	RCT (double blind)	Single	18	41	3 mo and 6 mo	NRS
Ozturk et al.^32^ (2012)	Turkey	MNC vs standard treatment	Peripheral blood mononuclear cells	Standard medication	RCT (double blind)	Single	20	20	3 mo and 6 mo	NRS
Sharma et al.^39^ (2021)	India	MNC vs standard treatment	Bone marrow mononuclear cells	Sham injection	RCT (double blind)	Single	27	29	3 mo and 6 mo	Binary
Teraa et al.^44^ (2015)	Netherlands	MNC vs standard treatment	Bone marrow mononuclear cells	Peripheral blood erythrocytes	RCT (double blind)	Single	81	79	3 mo and 6 mo	VAS
Belch et al.^3^ (2011)	Scotland	PGi vs standard treatment	Taprostene sodium	Normal saline	RCT (double blind)	Multi	74	37	1 mo	Binary
De Marchi et al.^9^ (2012)	Italy	PGi vs standard treatment	Propionyl-l-carnitine	Normal saline	RCT (double blind)	Single	24	24	Less than 2 wk	VAS
Dormandy^11^ (2000)	United Kingdom	PGi vs standard treatment	Higher-dose iloprost	Placebo	RCT (double blind)	Multi	207	207	1 y or more	VAS
Lawall et al.^27^ (2017)	Germany	PGi vs standard treatment	Intravenous alprostadil	Lactose infusion	RCT (double blind)	Multi	414	424	3 mo and 6 mo	VAS
Nehler et al.^30^ (2007)	USA	PGi vs standard treatment	Intravenous lipo-ecraprost	Placebo	RCT (double blind)	Multi	141	143	3 mo and 6 mo	Binary
Kanai et al.^24^ (2025)	Japan	scLido vs standard treatment	Subcutaneous lidocaine	Normal saline	RCT (double blind)	Single	8	8	Less than 2 wk	NRS
Spincemaille et al.^42^ (2000)	Netherlands	SCS vs standard treatment	Spinal cord stimulation	Medical treatment alone	RCT (double blind)	Multi	60	60	3 mo and 6 mo	VAS
Gonçalves et al.^15^ (2021)	Brazil	TENS vs standard treatment	Transcutaneous electric nerve Stimulation	Sham intervention	RCT (single blind)	Single	12	11	Less than 2 wk	VAS
Kusumanto et al.^25^ (2006)	Netherlands	VEGF vs standard treatment	Human vascular endothelial growth factor plasmid	Normal saline	RCT (double blind)	Multi	27	27	3 mo and 6 mo	VAS

Details of study design, participant characteristics, intervention type, comparator, and outcome measures for each included study.

HGF, hepatocyte growth factor gene therapy; Ket, ketamine; MNC, mononuclear cell therapy; PGi, prostaglandin infusion; RCT, randomised controlled trial; scLido, subcutaneous lidocaine; SCS, spinal cord stimulation; TENS, transcutaneous electrical nerve stimulation; VAS, Visual Analog Scale; VEGF, vascular endothelial growth factor gene therapy.

Interventions were categorised according to type of analgesic or adjunct therapy evaluated (Table 2).

**Table 2:** Summary of included studies by intervention type.

Intervention	Description	Study count and type	Treatment participants (n)	Control participants (n)
PGi vs standard treatment	Prostaglandin infusions for ischaemic pain relief	5 RCTs	860	835
MNC vs standard treatment	Mononuclear cell therapy	7 studies (5 RCTs, 2 cohort)	235	240
HGF vs standard treatment	Hepatocyte growth factor gene therapy	4 RCTs	441	249
Ket vs standard treatment	Intravenous ketamine infusion	1 RCT	18	17
TENS vs standard treatment	Transcutaneous electrical nerve stimulation	1 RCT	17	17
scLido vs standard treatment	Subcutaneous lidocaine infusion	1 RCT	8	8
VEGF vs standard treatment	Vascular endothelial growth factor gene therapy	1 RCT	27	27
SCS vs standard treatment	Spinal cord stimulation	1 RCT	60	60

Summary of included studies comparing different interventions with standard treatment for ischaemic pain in chronic limb–threatening ischaemia (CLTI). Study type and participant numbers (treatment and control) are provided for each intervention.

HGF, hepatocyte growth factor gene therapy; Ket, ketamine; MNC, mononuclear cell therapy; PGi, prostaglandin infusion; RCT, randomised controlled trial; scLido, subcutaneous lidocaine; SCS, spinal cord stimulation; TENS, transcutaneous electrical nerve stimulation; VEGF, vascular endothelial growth factor gene therapy.

Sample sizes for the treatment arms ranged from 8 participants (Kanai)^24^ to 414 participants (Lawall).^27^ The total number of participants in treatment arms and control arms were 1666 and 1453, respectively.

Most studies reported post-treatment pain scores at 3 and/or 6 months. For interventions evaluated by a single study, results were summarised narratively.

### 3.2. Risk of bias and quality assessment

Risk of bias was assessed for all included studies using the Cochrane Risk of Bias 2 (RoB 2) tool for RCTs (Table 3) and the NOS for nonrandomised studies (Table 4).

**Table 3:** Risk of bias assessment for included randomised controlled trials using the Cochrane Risk of Bias 2 tool.

Author	Year	Country	Groups	ROB2 domain 1	ROB2 domain 2	ROB2 domain 3	ROB2 domain 4	ROB2 domain 5	Overall
Anghel et al.^1^	2011	Romania	HGF vs standard treatment	1 = some concerns	0 = low risk	1 = some concerns	0 = low risk	1 = some concerns	1 = some concerns
Di et al.^10^	2022	China	HGF vs standard treatment	1 = some concerns	0 = low risk	1 = some concerns	0 = low risk	0 = low risk	1 = some concerns
Gu et al.^17^	2021	China	HGF vs standard treatment	0 = low risk	0 = low risk	1 = some concerns	0 = low risk	0 = low risk	1 = some concerns
Powell et al.^34^	2008	USA	HGF vs standard treatment	1 = some concerns	0 = low risk	0 = low risk	0 = low risk	0 = low risk	1 = some concerns
Mitchell and Fallon^29^	2002	United Kingdom	Ket vs standard treatment	0 = low risk	0 = low risk	0 = low risk	0 = low risk	0 = low risk	0 = low risk
Gupta et al.^18^	2013	India	MNC vs standard treatment	1 = some concerns	1 = some concerns	1 = some concerns	0 = low risk	0 = low risk	1 = some concerns
Lu et al.^28^	2011	China	MNC vs standard treatment	0 = low risk	0 = low risk	1 = some concerns	0 = low risk	1 = some concerns	1 = some concerns
Ozturk et al.^32^	2012	Turkey	MNC vs standard treatment	1 = some concerns	2 = high risk	1 = some concerns	2 = high risk	1 = some concerns	2 = high risk
Sharma et al.^39^	2021	India	MNC vs standard treatment	0 = low risk	0 = low risk	0 = low risk	0 = low risk	0 = low risk	0 = low risk
Teraa et al.^44^	2015	Netherlands	MNC vs standard treatment	0 = low risk	0 = low risk	0 = low risk	0 = low risk	0 = low risk	0 = low risk
Belch et al.^3^	2011	Scotland	PGi vs standard treatment	1 = some concerns	0 = low risk	0 = low risk	0 = low risk	1 = some concerns	1 = some concerns
De Marchi et al.^9^	2012	Italy	PGi vs standard treatment	0 = low risk	0 = low risk	0 = low risk	0 = low risk	1 = some concerns	1 = some concerns
Dormandy^11^	2000	United Kingdom	PGi vs standard treatment	0 = low risk	0 = low risk	1 = some concerns	0 = low risk	1 = some concerns	1 = some concerns
Lawall et al.^27^	2017	Germany	PGi vs standard treatment	1 = some concerns	0 = low risk	1 = some concerns	0 = low risk	0 = low risk	1 = some concerns
Nehler et al.^30^	2007	USA	PGi vs standard treatment	0 = low risk	0 = low risk	1 = some concerns	0 = low risk	0 = low risk	1 = some concerns
Kanai et al.^24^	2025	Japan	scLido vs standard treatment	1 = some concerns	0 = low risk	0 = low risk	0 = low risk	0 = low risk	1 = some concerns
Spincemaille et al.^42^	2000	Netherlands	SCS vs standard treatment	0 = low risk	2 = high risk	0 = low risk	1 = some concerns	0 = low risk	2 = high risk
Gonçalves et al.^15^	2021	Brazil	TENS vs standard treatment	0 = low risk	2 = high risk	0 = low risk	2 = high risk	0 = low risk	2 = high risk
Kusumanto et al.^25^	2006	Netherlands	VEGF vs standard treatment	0 = low risk	0 = low risk	1 = some concerns	0 = low risk	0 = low risk	1 = some concerns

Assessment of risk of bias across 5 RoB 2 domains: bias arising from the randomisation process, bias due to deviations from intended interventions, bias due to missing outcome data, bias in measurement of the outcome, and bias in selection of the reported result. Overall risk-of-bias judgements are provided for each trial.

HGF, hepatocyte growth factor gene therapy; Ket, ketamine; MNC, mononuclear cell therapy; PGi, prostaglandin infusion; RoB 2, Cochrane Risk of Bias 2 tool; scLido, subcutaneous lidocaine; SCS, spinal cord stimulation; TENS, transcutaneous electrical nerve stimulation; VEGF, vascular endothelial growth factor gene therapy.

**Table 4:** Risk of bias assessment for included nonrandomised studies using the Newcastle–Ottawa Scale.

Authors	Year	Country	Groups	Selection (max = 4)	Comparability (max = 2)	Outcome (max = 3)	Total (max = 9)
De Angelis et al.^8^	2015	Italy	MNC vs standard treatment	4	2	3	7
Gupta et al.^19^	2017	India	MNC vs standard treatment	4	2	2	6

Risk of bias assessment based on the NOS, which evaluates study quality across 3 domains: selection of study groups (maximum 4 points), comparability of groups (maximum 2 points), and ascertainment of the outcome for cohort studies or exposure for case–control studies (maximum 3 points). Scores out of a total of 9 points are reported, with higher scores indicating lower risk of bias.

max, maximum score; MNC, mononuclear cell therapy; NOS, Newcastle–Ottawa Scale.

Of the 19 RCTs,^1,3,8,10,11,15,17,18,24,25,27–30,32,34,39,42,44^ 3 were judged to be at *low risk* of bias across all RoB 2 domains,^29,39,44^ including bias arising from the randomisation process, deviations from intended interventions, missing outcome data, measurement of the outcome, and selection of the reported result. Thirteen trials were rated as having *some concerns* in one or more domains,^1,3,9–11,17,18,24,25,27,28,30,34,44^ most frequently related to deviations from intended interventions or issues in randomisation reporting. Last, 3 RCTs were judged to have high overall risk of bias.^15,32,42^

Two nonrandomised cohort studies were assessed with the NOS achieving scores of 7/9^8^ and 6/9,^19^ indicating a low risk of bias.

### 3.3. Three months and 6 months

After screening, 4 studies^8,17,19,32^ (3-month dataset) and 5 studies^17–19,27,28^ (6-month dataset) reported comparable post-treatment pain scores and were eligible for inclusion in the quantitative dataset. However, only the mononuclear cell therapy subgroup had ≥2 studies with harmonised continuous outcomes at each timepoint and was, therefore, meta-analysed. Treatment-arm participant totals across the included studies were 124 at 3 months and 536 at 6 months. Studies evaluated a range of interventions; subgroup meta-analyses were conducted by intervention category where there was sufficient data.

### 3.4. Mononuclear cell therapy vs standard treatment

Mononuclear cell therapy (MNC) exhibited a large statistically significant reduction in pain, but with high heterogeneity.

**Figure 2.**
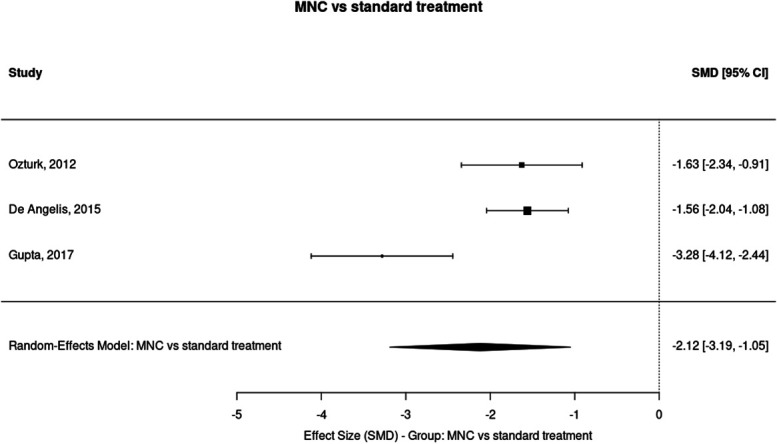
Forest plot of the effect of mononuclear cell (MNC) therapy vs standard treatment on ischaemic pain scores at 3 months. The random-effects meta-analysis showed a significant reduction in pain with MNC therapy compared with standard treatment (SMD = −2.12, 95% CI −3.19 to −1.05, *P* = 0.0001) across 3 studies (k = 3), with high heterogeneity (I^2^ = 86.9%). Negative SMD values indicate greater pain reduction in the treatment group. CI, confidence interval; SMD, standardised mean difference.

**Figure 3.**
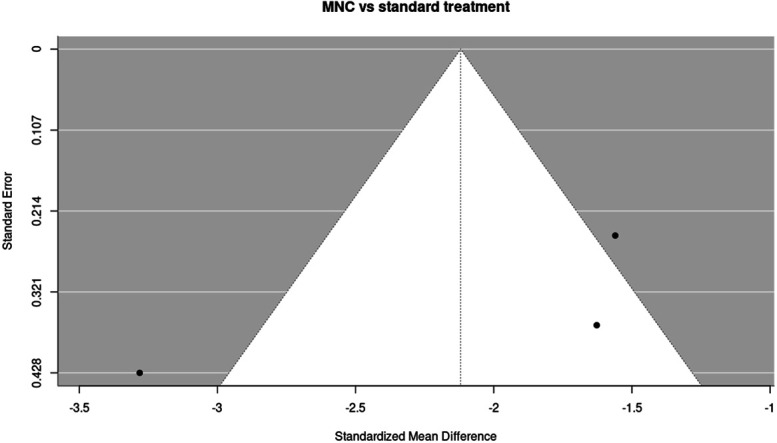
Funnel plot of studies comparing mononuclear cell (MNC) therapy vs standard treatment on ischaemic pain scores at 3 months. Funnel plot illustrating the distribution of effect sizes among the 3 studies (k = 3) included in the 3-month analysis of MNC therapy vs standard treatment. The plot is provided for visual assessment only; no statistical tests for funnel plot asymmetry were performed.

Three months (MNC vs standard treatment): random-effects estimate SMD = −2.12 (95% CI −3.19 to −1.05), *P* = 0.0001; I^2^ = 86.9% (k = 3) (Figs. 2 and 3).

Individual study estimates (Hedges g, 95% CI) contributing to the 3-month MNC pool:

(1) *Ozturk*^32^: g = −1.627 (95% CI −2.342, −0.912)
(2) *DeAngelis*^8^: g = −1.560 (95% CI −2.043, −1.077)
(3) *Gupta*^19^: g = −3.282 (95% CI −4.120, −2.443)

The funnel plot showed no clear visual evidence of asymmetry, although interpretation is limited by the small number of included studies.

**Figure 4.**
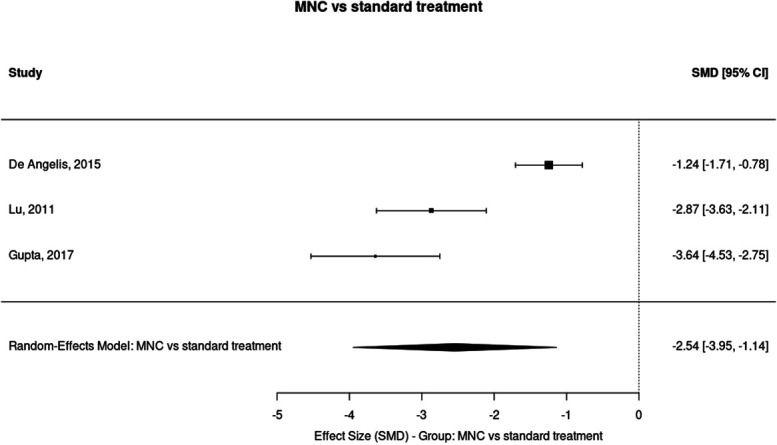
Forest plot of the effect of mononuclear cell (MNC) therapy vs standard treatment on ischaemic pain scores at 6 months. The random-effects meta-analysis demonstrated a significant reduction in pain with MNC therapy compared with standard treatment at 6 months (SMD = −2.54, 95% CI −3.95 to −1.14, *P* = 0.0004) across 3 studies (k = 3), with very high heterogeneity (I^2^ = 92.0%). Negative SMD values indicate greater pain reduction in the treatment group. CI, confidence interval; SMD, standardised mean difference.

**Figure 5.**
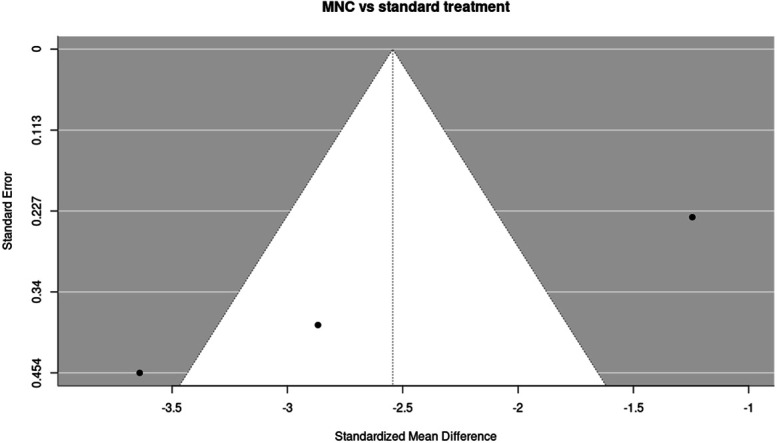
Funnel plot of studies comparing mononuclear cell (MNC) therapy vs standard treatment on ischaemic pain scores at 6 months. Funnel plot illustrating the distribution of effect sizes among the 3 studies (k = 3) included in the 6-month analysis of MNC therapy vs standard treatment. The plot is provided for visual assessment of potential asymmetry only; no statistical tests for funnel plot asymmetry were performed because of the small number of studies.

Six months (MNC vs standard treatment): random-effects estimate SMD = −2.54 (95% CI −3.95 to −1.14), *P* = 0.0004; I^2^ = 92.0% (k = 3) (Figs. 4 and 5).

Individual study estimates contributing to the 6-month MNC pool:

(1) *DeAngelis*^8^: g = −1.244 (95% CI −1.706, −0.783)
(2) *Lu*^28^: g = −2.868 (95% CI −3.626, −2.109)
(3) *Gupta*^19^: g = −3.641 (95% CI −4.530, −2.751)

The funnel plot showed possible visual asymmetry. Although interpretation is limited by the small number of studies, the presence of small-study effects cannot be ruled out and results should be interpreted cautiously.

### 3.5. Hepatocyte growth factor vs standard treatment

Hepatocyte growth factor (HGF) data were insufficient to run meta-analyses with continuous data, but across 3 RCTs^1,10,17^ assessing the binary outcome of pain presence at maximal change, HGF therapy was associated with a nonsignificant trend towards reduced pain compared with standard treatment (pooled log risk ratio = −0.46, 95% CI −1.01 to 0.09). Heterogeneity was moderate to high, driven by variability in effect sizes between studies (notably, one trial showed no effect while 2 favoured HGF).

### 3.6. Prostaglandin vs standard treatment

Prostaglandin infusion (PGi) data were insufficient to run meta-analyses with continuous data, but across 3 RCTs^3,27,30^ evaluating the binary outcome of pain presence at maximal change, PGi showed no significant effect compared with standard treatment (pooled log risk ratio = −0.05, 95% CI −0.17 to 0.07). Heterogeneity was negligible, with consistent null effects observed across trials. Therefore, there is no evidence to demonstrate it has analgesic potential in CLTI.

### 3.7. Single-study groups

#### 3.7.1. Pharmacological

##### 3.7.1.1. scLido vs sham

Kanai et al.^24^ randomised 8 participants to subcutaneous lidocaine (scLido) injection and 8 to normal saline injection (sham) injection. Lidocaine significantly decreased the numerical rating scale at 15 minutes postinjection from 10 (6, 10) (median [range]) to 2 (0, 10) (*P* < 0.001), and the differences between the 2 groups showed statistical significance (*P* = 0.009) in favour of the lidocaine group. No serious complications, including protracted bleeding, were reported.

##### 3.7.1.2. Ketamine vs standard treatment

Mitchell and Fallon^29^ randomised 18 participants to receive regular opioids plus ketamine and 17 to receive regular opioids plus placebo. Using the Brief Pain Inventory, the percentage of pain relief that the patients in the ketamine group attributed to their medication improved significantly from 50% immediately preinfusion to 65% 24 hours postinfusion and 69% 5 days postinfusion. Over the same period, the pain relief achieved by the placebo group declined from 58% preinfusion to 56% 24 hours postinfusion and then 50% relief 5 days later. This was statistically significant (*P* = 0.05). The ketamine group also showed a statistically significant difference 24 hours postinfusion of the effect of pain on their general activity (*P* = 0.03) and on their enjoyment of life (*P* = 0.004). In the study, haloperidol was used to counteract the adverse effects of low-dose ketamine and added to existing opioid analgesia. Using this regimen, side effects from the ketamine were reported as minimal.

#### 3.7.2. Nonpharmacological

##### 3.7.2.1. Vascular endothelial growth factor vs standard treatment

Kusumanto et al.^25^ randomised 27 participants to intramuscular injection of phVEGF165 (vascular endothelial growth factor [VEGF] gene-carrying plasmid) gene therapy and 27 to a placebo injection (0.9% NaCl) and measured pain on day 28, 72, and day 100 using Visual Analog Scale (VAS). There was a decrease in pain in 5 (18.5%) and 2 (7.4%) participants, respectively. This did not show statistical significance. There were no substantial adverse events in either group.

##### 3.7.2.2. Transcutaneous electrical nerve stimulation vs sham

In the Gonçalves et al. study,^15^ 34 patients were randomised: 17 to the transcutaneous electrical nerve stimulation (TENS) group and 17 to the sham group and measured patients' VAS in cm. The within-group analysis indicated a pain decrease in both groups (TENS group, from 7 to 3.9 cm, *P* < 0.0001, and the sham group from 5.8 to 3.2 cm, *P* < 0.0001). No statistically significant difference was verified between-groups (*P* = 0.5).

##### 3.7.2.3. Spinal cord stimulator vs standard treatment

Spincemaille et al.^42^ randomised 60 participants to spinal cord stimulator (SCS) and 60 patients to standard care and measured VAS pain at 1, 3, 6, 12, and 18 months. There was no statistically significant difference in pain relief nor quality of life between the 2 groups. However, patients in the SCS group used less narcotic and non-narcotic drugs (*P* < 0.002). Infection was reported in 3 cases and 3 batteries failed within 18 months. Because of these difficulties, 8 participants (13%) had suboptimal stimulation.

## 4. Discussion

Since its introduction, the WHO pain ladder—originally based primarily on evidence for oncological pain—has been widely employed for the management of all types of pain in institutions worldwide.^45^ Without procedures to revascularise or amputate ischaemic tissue, pain in patients with CLTI is often not managed adequately despite full use of the WHO pain ladder and is associated with significant systemic adverse effects. This systematic review set out to assess the evidence for analgesic strategies outside of the WHO pain ladder.

The findings of this review should be interpreted within the broader evidence base for pain management in CLTI. Previous reviews in this field have predominantly examined revascularisation strategies or perfusion-enhancing therapies, with comparatively little emphasis on analgesic approaches. Evidence for pain-directed interventions in CLTI remains sparse, methodologically variable, and unevenly distributed across intervention types. Overall, the limited and inconsistent nature of the evidence made it important to collate and assess the studies that were available.

### 4.1. Mononuclear cell therapy

Across the included studies, mononuclear cell therapy demonstrated the largest effect sizes for pain reduction at both 3 and 6 months, but high heterogeneity and possible small-study effects were observed. The applicability of the MNC trial findings to real-world CLTI populations is highly limited by their extensive exclusion criteria, which omitted patients with common comorbidities such as diabetes; severe cardiovascular disease; chronic kidney disease; prior stroke; recent malignancy; and active infection. These criteria effectively exclude a substantial proportion of patients typically encountered in clinical practice. Consequently, MNC is unlikely to be a pragmatic analgesic option for CLTI, as its use would be restricted to a small, highly selected subset of patients.

### 4.2. Hepatocyte growth factor

Hepatocyte growth factor therapy showed a nonsignificant trend towards pain reduction, with high heterogeneity. This, combined with prohibitive exclusion criteria—poor inflow vessels; severe renal impairment; drug or alcohol abuse; and recent myocardial infarction or stroke, mean it does not demonstrate potential as a CLTI ischaemic pain agent.

### 4.3. Prostaglandin

Prostaglandin infusion showed no meaningful effect on pain relief and also excluded a large proportion of patients seen in practice. Exclusion criteria included in these trials included patients taking any antiplatelet other than aspirin and those with a planned amputation or revascularisation within the next 2 weeks, which means the results would not be applicable to a significant proportion of patients with CLTI.

### 4.4. Single-study groups

Of the single-study interventions, subcutaneous lidocaine and ketamine both showed short-term analgesic benefit with acceptable safety, whereas transcutaneous electrical nerve stimulation, vascular endothelial growth factor therapy, and spinal cord stimulation did not produce statistically significant improvements in pain outcomes. Similar results for ketamine and intravenous lidocaine have been previously reported in a 2017 systematic review on pharmacological analgesic therapies for CLTI pain.^26^

Regarding the generalisability of the results from the SCS trial,^42^ extensive exclusion criteria—including intractable infections of the ulceration or gangrenous area; concomitant disease limiting life expectancy to less than 1 year; the presence of a cardiac pacemaker; and inadequate patient compliance—mean that the results of this study are not applicable to a large proportion of patients with CLTI.

### 4.5. Safety, quality of life, and methodological considerations

Safety and quality-of-life outcomes are important considerations when interpreting these findings. Adverse event reporting varied widely and was often incomplete, limiting conclusions about comparative safety. Where reported, most interventions appeared well tolerated, although small samples and selective populations may have missed uncommon or longer-term harms. Quality-of-life outcomes were rarely measured using validated tools, despite the substantial burden of CLTI. Future studies should incorporate standardised safety reporting and validated quality-of-life measures alongside pain outcomes to better reflect the real-world impact of potential treatments.

Several methodological issues across the included studies also warrant consideration. Pain outcomes were measured using a range of scales, reported at inconsistent timepoints, and often lacked complete variance data, limiting comparability and preventing broader quantitative synthesis. Follow-up durations varied, with relatively few studies assessing longer-term analgesic benefit. Many trials also used restrictive eligibility criteria and had small sample sizes, reflecting the challenges of research in a frail population with high morbidity and mortality.

Future studies should use standardised pain outcome measures, report complete summary statistics, and align follow-up timepoints to allow comparison across interventions. In addition, greater involvement of patients and the public at the design stage is essential to ensure that trials capture outcomes that matter most to people living with CLTI, such as quality of life, functional ability, sleep, and how ischaemic pain affects daily life.

### 4.6. Limitations

This review has several limitations. Only a small number of studies were eligible for inclusion, and substantial variation in trial design, outcome measures, and reporting contributed to heterogeneity. Many studies applied restrictive eligibility criteria, limiting generalisability to the wider CLTI population. Incomplete reporting of pain data, safety outcomes, and quality-of-life measures further constrained the strength of the conclusions. Publication bias cannot be excluded given the small evidence base.

## 5. Conclusion

Based on the findings of this systematic review, no recommendations can be made for the routine use of any analgesic method or adjunct outside of standard care for CLTI. Mononuclear cell therapy demonstrated the largest effect sizes for pain reduction, but high heterogeneity, evidence of small-study effects, and highly restrictive exclusion criteria significantly limit generalisability to typical CLTI populations, making it an impractical management strategy. Limited evidence from single small trials suggests that ketamine and subcutaneous lidocaine may have potential as analgesic options, but robust, high-quality studies are required to confirm their efficacy and safety. Further research into novel analgesic agents for the treatment of CLTI is urgently needed.

## Disclosures

The authors have no conflict of interest to declare.

## Supplemental digital content

Supplemental digital content associated with this article can be found online at http://links.lww.com/PR9/A392.

## Supplementary Material

SUPPLEMENTARY MATERIAL
