# Supplementary material for: Analgesic strategies for ischaemic pain in chronic limb–threatening ischaemia: a systematic review and meta-analysis
Source: Pain Rep. 2026 Mar 10;11(2):e1426. doi: 10.1097/PR9.0000000000001426 (PMC12978830; doi:10.1097/PR9.0000000000001426)
Supplement: SUPPLEMENTARY MATERIAL [file painreports-11-e1426-s001.pdf]

Supplementary Figure 1. Database search strategy for the systematic review of analgesic interventions for ischaemic pain in chronic limb-threatening ischaemia (CLTI).

CONTENTS

Overview ... 2

STRATEGY: Ovid MEDLINE(R) ALL <1946 to February 28, 2025> (run 03/03/2025) ... 3

STRATEGY: OVID Embase <1974 to 2025 February 28> (run 03/03/2025) ... 6

STRATEGY: EBSCO CINAHL RUN 03/03/2025 ... 9

STRATEGY: COCHRANE: Date Run: 03/03/2025 12:10:22 ... 13

Overview

DATABASE	Results retrieved	Total	Duplicates	Total after deduplication
Ovid Medline	141	631	234	397
Ovid Embase	373			
EBSCO Cinahl	16			
Cochrane	101			
Web of Science				

STRATEGY: Ovid MEDLINE(R) ALL <1946 to February 28, 2025> (run 03/03/2025)

1	Analgesia/	22631
2	analgesia.ti,ab.	75628
3	"pain management".ti,ab.	36763
4	Pain Management/	44368
5	"pain relief".ti,ab.	40961
6	opioid.ti,ab.	107868
7	Analgesics, Opioid/	65740
8	NSAID*s.ti,ab.	24124
9	"Non-steroidal anti-inflammatory drugs".ti,ab.	14345
10	Anti-Inflammatory Agents, Non-Steroidal/	73875
11	"nerve block*".ti,ab.	16106
12	Nerve Block/	22984
13	"spinal cord stimulation".ti,ab.	4460
14	Spinal Cord Stimulation/	2193
15	1 or 2 or 3 or 4 or 5 or 6 or 7 or 8 or 9 or 10 or 11 or 12 or 13 or 14	369646
16	Ischemia/	56895
17	ischemia.ti,ab.	215029
18	ischaemia.ti,ab.	31979
19	ischemic.ti,ab.	265480
20	ischaemic.ti,ab.	46825
21	16 or 17 or 18 or 19 or 20	488771
22	"chronic limb-threatening isch*emia".ti,ab.	1452
23	CLTI.ti,ab.	1012
24	"critical limb isc*emia".ti,ab.	4865
25	CLI.ti,ab.	3411
26	"peripheral arter* disease".ti,ab.	19400

27 PAD.ti,ab. 32661

28 "peripheral vascular disease".ti,ab. 10168

29 PVD.ti,ab. 3625

30 "peripheral arterial occlusive disease".ti,ab. 1958

31 "peripheral obliterative arteriopathy".ti,ab. 13

32 "diabetic foot ulcer*".ti,ab. 7107

33 DFU.ti,ab. 2436

34 22 or 23 or 24 or 25 or 26 or 27 or 28 or 29 or 30 or 31 or 32 or 33 70278

35 (Randomized Controlled Trial or Controlled Clinical Trial or Pragmatic Clinical Trial or Clinical Study or Adaptive Clinical Trial or Equivalence Trial).pt. 731814

36 (Clinical Trial or Clinical Trial, Phase I or Clinical Trial, Phase II or Clinical Trial, Phase III or Clinical Trial, Phase IV or Clinical Trial Protocol).pt. 625733

37 Multicenter Study.pt. 364936

38 Clinical Studies as Topic/ 869

39 exp Clinical Trial/ or exp Clinical Trials as Topic/ or Clinical Trial Protocol/ or Clinical Trial Protocols as Topic/ or exp "Clinical Trial (topic)"/ 1341950

40 Multicenter Study/ or Multicenter Studies as Topic/ or "Multicenter Study (topic)"/ 386961

41 Randomization/108141

42 Random Allocation/ 108141

43 Double-Blind Method/ 182849

44 Double-Blind Studies/ 182849

45 Single-Blind Method/ 34661

46 Single-Blind Studies/ 34661

47 Placebos/ 36068

48 Control Groups/2145

49 Control Group/ 2145

50 Cross-Over Studies/ or Crossover Procedure/ 58545

51 (random* or sham or placebo*).ti,ab,hw,kf. 2000141

52 ((singl* or doubl*) adj (blind* or dumm* or mask*)).ti,ab,hw,kf. 283142

53 ((tripl* or trebl*) adj (blind* or dumm* or mask*)).ti,ab,hw,kf. 2045

54 (control* adj3 (study or studies or trial* or group*)).ti,ab,hw,kf. 2089908

55	(clinical adj3 (study or studies or trial*)).ti,ab,hw,kf.	1543353

56	(Nonrandom* or non random* or non-random* or quasi-random* or quasirandom*).ti,ab,hw,kf.	60988

57	(phase adj6 (study or studies or trial*)).ti,ab,hw,kf.	234545

58	((crossover or cross-over) adj3 (study or studies or trial*)).ti,ab,hw,kf.	81658

59	((multicent* or multi-cent*) adj3 (study or studies or trial*)).ti,ab,hw,kf.	449979

60	allocated.ti,ab,hw.	93779

61	((open label or open-label) adj5 (study or studies or trial*)).ti,ab,hw,kf.	50670

62	((equivalence or superiority or non-inferiority or noninferiority) adj3 (study or studies or trial*)).ti,ab,hw,kf.	14630

63	(pragmatic study or pragmatic studies).ti,ab,hw,kf.	696

64	((pragmatic or practical) adj3 trial*).ti,ab,hw,kf.	9176

65	((quasiexperimental or quasi-experimental) adj3 (study or studies or trial*)).ti,ab,hw,kf. 15255

66	trial.ti,kf.	355537

67	35 or 36 or 37 or 38 or 39 or 40 or 41 or 42 or 43 or 44 or 45 or 46 or 47 or 48 or 49 or 50 or 51 or 52 or 53 or 54 or 55 or 56 or 57 or 58 or 59 or 60 or 61 or 62 or 63 or 64 or 65 or 66 4183825

68	exp animals/	27870054

69	exp animal experimentation/	10666

70	exp models animal/	674985

71	exp animal experiment/	10666

72	exp vertebrate/	27086603

73	68 or 69 or 70 or 71 or 72	27872034

74	exp humans/	22558552

75	73 not 74	5313482

76	67 not 75	3759388

77	15 and 21 and 34 and 76	141

STRATEGY: OVID EMBASE <1974 TO 2025 FEBRUARY 28> (RUN 03/03/2025)

1	Analgesia/	169852
2	analgesia.ti,ab.	106865
3	"pain management".ti,ab.	53674
4	Pain Management/	169852
5	"pain relief".ti,ab.	61572
6	opioid.ti,ab.	146476
7	Analgesics, Opioid/	29525
8	NSAID*s.ti,ab.	43163
9	"Non-steroidal anti-inflammatory drugs".ti,ab.	20230
10	Anti-Inflammatory Agents, Non-Steroidal/	155006
11	"nerve block*".ti,ab.	23200
12	Nerve Block/	43576
13	"spinal cord stimulation".ti,ab.	8796
14	Spinal Cord Stimulation/	10804
15	1 or 2 or 3 or 4 or 5 or 6 or 7 or 8 or 9 or 10 or 11 or 12 or 13 or 14	568210
16	Ischemia/	91335
17	ischemia.ti,ab.	304666
18	ischaemia.ti,ab.	42719
19	ischemic.ti,ab.	410992
20	ischaemic.ti,ab.	68914
21	16 or 17 or 18 or 19 or 20	728460
22	"chronic limb-threatening isch*emia".ti,ab.	2106
23	CLTI.ti,ab.	1561
24	"critical limb isc*emia".ti,ab.	7985
25	CLI.ti,ab.	5750
26	"peripheral arter* disease".ti,ab.	31531
27	PAD.ti,ab.	50848
28	"peripheral vascular disease".ti,ab.	16464

29 PVD.ti,ab. 6001

30 "peripheral arterial occlusive disease".ti,ab. 2833

31 "peripheral obliterative arteriopathy".ti,ab. 7

32 "diabetic foot ulcer*".ti,ab. 9756

33 DFU.ti,ab. 3258

34 22 or 23 or 24 or 25 or 26 or 27 or 28 or 29 or 30 or 31 or 32 or 33 108776

35 (Randomized Controlled Trial or Controlled Clinical Trial or Pragmatic Clinical Trial or Clinical Study or Adaptive Clinical Trial or Equivalence Trial).pt. 0

36 (Clinical Trial or Clinical Trial, Phase I or Clinical Trial, Phase II or Clinical Trial, Phase III or Clinical Trial, Phase IV or Clinical Trial Protocol).pt. 0

37 Multicenter Study.pt. 0

38 Clinical Studies as Topic/ 167932

39 exp Clinical Trial/ or exp Clinical Trials as Topic/ or Clinical Trial Protocol/ or Clinical Trial Protocols as Topic/ or exp "Clinical Trial (topic)"/ 2440394

40 Multicenter Study/ or Multicenter Studies as Topic/ or "Multicenter Study (topic)"/ 457471

41 Randomization/100317

42 Random Allocation/ 100317

43 Double-Blind Method/ 228902

44 Double-Blind Studies/ 228902

45 Single-Blind Method/ 58211

46 Single-Blind Studies/ 58211

47 Placebos/ 423710

48 Control Groups/109455

49 Control Group/ 109455

50 Cross-Over Studies/ or Crossover Procedure/ 81324

51 (random* or sham or placebo*).ti,ab,hw,kf. 2795519

52 ((singl* or doubl*) adj (blind* or dumm* or mask*)).ti,ab,hw,kf. 389091

53 ((tripl* or trebl*) adj (blind* or dumm* or mask*)).ti,ab,hw,kf. 2636

54 (control* adj3 (study or studies or trial* or group*)).ti,ab,hw,kf. 11948626

55 (clinical adj3 (study or studies or trial*)).ti,ab,hw,kf. 7405972

56 (Nonrandom* or non random* or non-random* or quasi-random* or quasirandom*).ti,ab,hw,kf. 77262

57 (phase adj6 (study or studies or trial*)).ti,ab,hw,kf. 506702

58 ((crossover or cross-over) adj3 (study or studies or trial*)).ti,ab,hw,kf. 74022

59 ((multicent* or multi-cent*) adj3 (study or studies or trial*)).ti,ab,hw,kf. 579432

60 allocated.ti,ab,hw. 120718

61 ((open label or open-label) adj5 (study or studies or trial*)).ti,ab,hw,kf. 97317

62 ((equivalence or superiority or non-inferiority or noninferiority) adj3 (study or studies or trial*)).ti,ab,hw,kf. 21521

63 (pragmatic study or pragmatic studies).ti,ab,hw,kf. 1082

64 ((pragmatic or practical) adj3 trial*).ti,ab,hw,kf. 10336

65 ((quasiexperimental or quasi-experimental) adj3 (study or studies or trial*)).ti,ab,hw,kf. 23337

66 trial.ti,kf. 491908

67 35 or 36 or 37 or 38 or 39 or 40 or 41 or 42 or 43 or 44 or 45 or 46 or 47 or 48 or 49 or 50 or 51 or 52 or 53 or 54 or 55 or 56 or 57 or 58 or 59 or 60 or 61 or 62 or 63 or 64 or 65 or 66 16215575

68 exp animals/ 33324612

69 exp animal experimentation/ 3314635

70 exp models animal/ 1938176

71 exp animal experiment/ 3314635

72 exp vertebrate/ 32699239

73 68 or 69 or 70 or 71 or 72 33531531

74 exp humans/ 27601338

75 73 not 74 5930193

76 67 not 75 13480667

77 15 and 21 and 34 and 76 373

STRATEGY: EBSCO CINAHL RUN 03/03/2025

#	Query	Results
S77	S15 AND S21 AND S34 AND S76	16
S76	S67 NOT S75	847,680
S75	S67 NOT S74	326,809
S74	(MH "Human")	2,865,569
S73	S68 OR S69 OR S70 OR S71 OR S72	3,096,826
S72	(MH "Vertebrates+")	3,081,761
S71	(MH "Animal Studies")	153,489
S70	(MH "Models, Biological")	72,891
S69	(MH "Animals, Laboratory")	713
S68	(MH "Animals+")	100,490
S67	S35 OR S36 OR S37 OR S38 OR S39 OR S40 OR S41 OR S42 OR S43 OR S44 OR S45 OR S46 OR S47 OR S48 OR S49 OR S50 OR S51 OR S52 OR S53 OR S54 OR S55 OR S56 OR S57 OR S58 OR S59 OR S60 OR S61 OR S62 OR S63 OR S64 OR S65 OR S66	1,174,489
S66	TI trial	203,419
S65	TI (((quasiexperimental or quasi-experimental) n3 (study or studies or trial*))) OR AB (((quasiexperimental or quasi-experimental) n3 (study or studies or trial*)))	10,994
S64	TI (((pragmatic or practical) n3 trial*)) AND AB (((pragmatic or practical) n3 trial*))	785
S63	TI ((pragmatic study or pragmatic studies)) OR AB ((pragmatic study or pragmatic studies))	291
S62	TI (((equivalence or superiority or non-inferiority or noninferiority) n3 (study or studies or trial*))) AND AB (((equivalence or superiority or non-inferiority or noninferiority) n3 (study or studies or trial*)))	911
S61	TI (((open label or open-label) n5 (study or studies or trial*))) OR AB (((open label or open-label) n5 (study or studies or trial*)))	17,209
S60	TI allocated OR AB allocated	26,249
S59	TI (((multicent* or multi-cent*) n3 (study or studies or trial*))) OR AB (((multicent* or multi-cent*) n3 (study or studies or trial*)))	59,167
S58	TI (((crossover or cross-over) n3 (study or studies or trial*))) OR AB (((crossover or cross-over) n3 (study or studies or trial*)))	16,403

S57	TI ((phase n6 (study or studies or trial*))) OR AB ((phase n6 (study or studies or trial*)))	61,951
S56	TI ((Nonrandom* or non random* or non-random* or quasi-random* or quasirandom*)) OR AB ((Nonrandom* or non random* or non-random* or quasi-random* or quasirandom*))	16,000
S55	TI ((clinical n3 (study or studies or trial*))) OR AB ((clinical n3 (study or studies or trial*)))	226,236
S54	TI ((control* n3 (study or studies or trial* or group*))) OR AB ((control* n3 (study or studies or trial* or group*)))	392,829
S53	TI (((tripl* or trebl*) n1 (blind* or dumm* or mask*))) OR AB (((tripl* or trebl*) n1 (blind* or dumm* or mask*)))	839
S52	TI (((singl* or doubl*) n1 (blind* or dumm* or mask*))) OR AB (((singl* or doubl*) n1 (blind* or dumm* or mask*)))	60,891
S51	TI ((random* or sham or placebo*)) OR AB ((random* or sham or placebo*))	498,787
S50	MH ("Cross-Over Studies" or "Crossover Procedure")	0
S49	MH Control Group	15,352
S48	MH Control Groups	0
S47	MH Placebos	14,258
S46	MH Single-Blind Studies	16,256
S45	MH Single-Blind Method	0
S44	MH Double-Blind Studies	54,512
S43	MH Double-Blind Method	0
S42	MH Random Allocation	0
S41	MH Randomization	0
S40	Multicenter Study/ or Multicenter Studies as Topic/ or "Multicenter Study (topic)"/	360,534
S39	exp Clinical Trial/ or exp Clinical Trials as Topic/ or Clinical Trial Protocol/ or Clinical Trial Protocols as Topic/ or exp "Clinical Trial (topic)"/	367
S38	Clinical Studies as Topic/	21,780
S37	PT ((Clinical Trial or Clinical Trial, Phase I or Clinical Trial, Phase II or Clinical Trial, Phase III or Clinical Trial, Phase IV or Clinical Trial Protocol))	114,285
S36	PT ((Clinical Trial or Clinical Trial, Phase I or Clinical Trial, Phase II or Clinical Trial, Phase III or Clinical Trial, Phase IV or Clinical Trial Protocol))	114,285

S35	PT ((Randomized Controlled Trial or Controlled Clinical Trial or Pragmatic Clinical Trial or Clinical Study or Adaptive Clinical Trial or Equivalence Trial))	161,211
S34	S22 OR S23 OR S24 OR S25 OR S26 OR S27 OR S28 OR S29 OR S30 OR S31 OR S32 OR S33	18,990
S33	TI DFU OR AB DFU	1,329
S32	TI "diabetic foot ulcer*" OR AB "diabetic foot ulcer*"	3,955
S31	TI "peripheral obliterative arteriopathy" OR AB "peripheral obliterative arteriopathy"	0
S30	TI "peripheral arterial occlusive disease" OR AB "peripheral arterial occlusive disease"	278
S29	TI PVD OR AB PVD	578
S28	TI "peripheral vascular disease" OR AB "peripheral vascular disease"	2,259
S27	TI PAD OR AB PAD	7,478
S26	TI "peripheral arter* disease" OR AB "peripheral arter* disease"	5,520
S25	TI CLI OR AB CLI	601
S24	TI "critical limb isc*emia" OR AB "critical limb isc*emia"	1,141
S23	TI CLTI OR AB CLTI	140
S22	TI "chronic limb-threatening isch*emia" OR AB "chronic limb-threatening isch*emia"	261
S21	S16 OR S17 OR S18 OR S19 OR S20	94,520
S20	TI ischemic OR AB ischemic	61,105
S19	TI ischaemic OR AB ischaemic	61,105
S18	TI ischaemia OR AB ischaemia	31,346
S17	TI ischemia OR AB ischemia	31,346
S16	Ischemia/	58,755
S15	S1 OR S2 OR S3 OR S4 OR S5 OR S6 OR S7 OR S8 OR S9 OR S10 OR S11 OR S12 OR S13 OR S14	131,296
S14	(MH "Spinal Cord Stimulation")	402
S13	TI ("spinal cord stimulation") OR AB ("spinal cord stimulation")	1,168
S12	(MH "Nerve Block")	10,551
S11	TI "nerve block*" OR AB "nerve block*"	5,337
S10	(MH "Antiinflammatory Agents, Non-Steroidal")	15,082
S9	TI "Non-steroidal anti-inflammatory drugs" OR AB "Non-steroidal anti-inflammatory drugs"	2,176

S8	TI NSAID*s OR AB NSAID*s	5,181
S7	(MH "Analgesics, Opioid")	25,362
S6	TI opioid OR AB opioid	46,839
S5	TI "pain relief" OR AB "pain relief"	15,272
S4	(MH "Pain Management")	18,942
S3	TI "pain management" OR AB "pain management"	21,178
S2	TI analgesia OR AB analgesia	20,742
S1	(MH "Analgesia")	7,490

STRATEGY: COCHRANE: DATE RUN: 03/03/2025 12:10:22

ID	Search	Hits
#1	MeSH descriptor: [Analgesia] this term only	2872
#2	analgesia:ti,ab	45597
#3	MeSH descriptor: [Pain Management] this term only	6049
#4	"pain management":ti,ab	11502
#5	"pain relief":ti,ab	20856
#6	MeSH descriptor: [Analgesics, Opioid] this term only	10666
#7	opioid:ti,ab	24655
#8	NSAID's:ti,ab	4045
#9	"non steroidal anti-inflammatory drugs":ti,ab	2664
#10	MeSH descriptor: [Anti-Inflammatory Agents, Non-Steroidal] this term only	8090
#11	nerve block:ti,ab	16667
#12	MeSH descriptor: [Nerve Block] this term only	5204
#13	"spinal cord stimulation":ti,ab	1073
#14	MeSH descriptor: [Spinal Cord Stimulation] this term only	227
#15	#1 OR #2 OR #3 OR #4 OR #5 OR #6 OR #7 OR #8 OR #9 OR #10 OR #11 OR #12 OR #13 OR #14	101573
#16	MeSH descriptor: [Ischemia] this term only	2949
#17	ischemia:ti,ab	16248
#18	ischaemia:ti,ab	16248
#19	ischemic:ti,ab	36567
#20	ischaemic:ti,ab	36567
#21	#17 OR #18 OR #19 OR #20	47738
#22	"chronic limb-threatening ischaemia":ti,ab	151
#23	CLTI:ti,ab	102
#24	"critical limb ischemia":ti,ab	816
#25	CLI:ti,ab	644
#26	peripheral arterial disease:ti,ab	5176
#27	PAD:ti,ab	6751

#28 "peripheral vascular disease":ti,ab 1270

#29 PVD:ti,ab 492

#30 peripheral arterial occlusive disease:ti,ab 1291

#31 peripheral obliterative arteriopathy 8

#32 diabetic foot ulcer*:ti,ab 2992

#33 DFU 536

#34 #22 OR #23 OR #24 OR #25 OR #26 OR #27 OR #28 OR #29 OR #30 OR #31 OR #32 OR #33 15436

#35 (Randomized Controlled Trial or Controlled Clinical Trial or Pragmatic Clinical Trial or Clinical Study or Adaptive Clinical Trial or Equivalence Trial):ti,ab 758212

#36 (Clinical Trial or Clinical Trial, Phase I or Clinical Trial, Phase II or Clinical Trial, Phase III or Clinical Trial, Phase IV or Clinical Trial Protocol):ti,ab 400012

#37 Multicenter Study:ti,ab 144870

#38 clinical studies:ti,ab 198755

#39 MeSH descriptor: [Clinical Trial] explode all trees42

#40 MeSH descriptor: [Multicenter Studies as Topic] explode all trees8910

#41 MeSH descriptor: [Random Allocation] explode all trees 25549

#42 MeSH descriptor: [Double-Blind Method] explode all trees 166822

#43 MeSH descriptor: [Single-Blind Method] explode all trees 26903

#44 MeSH descriptor: [Placebos] explode all trees 26637

#45 MeSH descriptor: [Control Groups] explode all trees 334

#46 MeSH descriptor: [Cross-Over Studies] explode all trees 47511

#47 (random* or sham or placebo):ti,ab 1409767

#48 ((singl* or doubl*) NEAR (blind* or dumm* or mask*)):ti,ab 343413

#49 ((tripl* or trebl*) NEAR (blind* or dumm* or mask*)):ti,ab 3612

#50 (control*) NEAR/3 (study or studies or trial* or group*):ti,ab 771413

#51 clinical NEAR/3 (study or studies or trial*):ti,ab 360489

#52 (Nonrandom* or non random* or non-random* or quasi-random* or quasirandom*):ti,ab 202666

#53 (phase NEAR/6 (study or studies or trial*)):ti,ab 159507

#54 ((crossover or cross-over) NEAR/3 (study or studies or trial*)):ti,ab 73437

#55 ((multicent* or multi-cent*) NEAR/3 (study or studies or trial*)):ti,ab 97590

#56 allocated:ti,ab 83905

#57 ((open label or open-label) NEAR/5 (study or studies or trial*)):ti,ab 83291

#58 ((equivalence or superiority or non-inferiority or noninferiority) NEAR/3 (study or studies or trial*)):ti,ab 17389

#59 (pragmatic study or pragmatic studies):ti,ab 10209

#60 ((pragmatic or practical) NEAR/3 trial):ti,ab 7046

#61 ((quasiexperimental or quasi-experimental) NEAR/3 (study or studies or trial*)):ti,ab 4291

#62 trial:ti,ab 834259

#63 #35 OR #36 OR #37 OR #38 OR #39 OR #40 OR #41 OR #42 OR #43 OR #44 OR #45 OR #46 OR #47 OR #48 OR #49 OR #50 OR #51 OR #52 OR #53 OR #54 OR #55 OR #56 OR #57 OR #58 OR #59 OR #60 OR #61 OR #62 1731261

#64 MeSH descriptor: [Animal Experimentation] explode all trees 11

#65 MeSH descriptor: [Models, Animal] explode all trees 1155

#66 MeSH descriptor: [Vertebrates] explode all trees 887473

#67 MeSH descriptor: [Child] explode all trees 81439

#68 MeSH descriptor: [Infant] explode all trees 45268

#69 MeSH descriptor: [Adolescent] explode all trees 135760

#70 #64 OR #65 OR #66 887485

#71 MeSH descriptor: [Humans] explode all trees 884049

#72 MeSH descriptor: [Adult] explode all trees 616194

#73 #70 NOT #71 3436

#74 #63 NOT #73 1728124

#75 #15 AND #21 AND #34 AND #74 101

Legend: We searched MEDLINE, EMBASE, the Cochrane Library, Web of Science, and CINAHL from 2000 to 2025 without language restrictions. The strategy combined indexed terms and free-text keywords for “analgesia,” “ischaemic pain,” and “chronic limb-threatening ischaemia,” adapted for each database. Additional sources included reference lists of eligible studies and relevant reviews, as well as direct contact with authors and experts in the field.
